# Yeast screening platform identifies FDA-approved drugs that reduce Aβ oligomerization

**DOI:** 10.15698/mic2016.03.482

**Published:** 2016-03-03

**Authors:** Triana Amen, Daniel Kaganovich

**Affiliations:** 1 Department of Cell and Developmental Biology, Alexander Silberman Institute of Life Sciences, Hebrew University of Jerusalem, Jerusalem 91904, Israel.; 2 Alexander Grass Center for Bioengineering, Hebrew University of Jerusalem, Jerusalem, Israel.

**Keywords:** yeast, drug, FDA, Alzheimer’s, screen, amyloid beta

The older the average person alive today becomes, the more instances of neurodegeneration are observed world-wide. Alzheimer's disease is the most common neurodegenerative disorder preferentially affecting older individuals with 26.6 million cases recorded in 2006. It is estimated that worldwide prevalence will rise to 100 million cases by 2050 1. There is currently no effective treatment nor preventative therapy for Alzheimer's disease, and no definitive diagnosis besides post-mortem pathology. Diagnosis is based on the presence of intracellular inclusions of hyperphosphorylated microtubule associated protein tau and extracellular plaques consisting of amyloid beta (Aβ) peptide 2. Aβ is a small peptide 40-42 aa in length, formed via amyloid precursor protein (APP) cleavage that results in Aβ release into the extracellular space. Aβ is normally observed circulating in the cerebrospinal fluid of mammals, and is produced mostly in the central nervous system 3. Although Aβ aggregates are the major pathological hallmark of Alzheimer’s disease, the mechanisms of Aβ induced neurotoxicity is not well understood, and even less is known about the physiological function of Aβ peptide. Absence of APP results in embryonic development defects due to irregular migration of cerebral cortex neurons 4. Recent work also indicates that Aβ peptide concentrations in the CNS modulate synaptic transmission and synaptic hyperactivity via direct binding to APP 5.

In addition to the pathological connection between Aβ deposition and Alzheimer’s, a genetic connection has been mapped as well. Multiple mutations in APP and its cleaving enzymes increase the risk of Alzheimer disease onset 6,7,8. Some mutations alter the cleavage of APP, resulting in a shifted ratio of Aβ1-42 to Aβ1-40, thus increasing the proportion of the more aggregation-prone species. Other mutations affect the aggregation propensity of the Aβ1-40/42 peptide itself 9. As with another aggregation-prone disease associated protein, α-synuclein in Parkinson’s disease, an increase in Aβ production results in its aggregation and the early onset of Alzheimer’s disease 10.

While most models of Aβ cellular pathology assume that toxicity stems from its aggregation propensity 11, there has been vigorous debate about whether the toxicity stems mostly from extracellular high-molecular weight amyloid plaques, or mostly from the low molecular weight oligomers 12,13,14. Aβ can be re-incorporated into the cytoplasm after extra-cellular cleavage, and much evidence has accumulated over the past several years that favors the small intracellular oligomers as the toxic aggregate species 15. Particularly convincing are seminal studies in simple models of disease: *C. elegans* and mice, demonstrating a link between aging, insulin signaling, and toxicity driven by low molecular weight oligomers of Aβ 16,17,18. Another study, modeling Alzheimer's disease in mice, showed that cognitive impairment precedes mature fibrillar deposits 19.

Due to the multifaceted and multifactorial nature of Alzheimer’s physiology, no single model can fully recapitulate disease. Mice are currently the model system that most closely resembles human beings while still being capable of exhibiting features of aging on a time-scale in line with the duration of a typical PhD or postdoc. Mice can also be scored for learning and memory defects, as well as motor neuron function. However, it is equally the case that a mouse that is artificially expressing extremely high amounts of Aβ in its brain will not accurately recapitulate the memory neuronal circuits of a 75-year-old human being. At the same time, mammalian models are sometimes less tractable and may offer less molecular and cellular detail of pathology and toxic events.

Simpler models of Aβ toxicity have been exploited with tremendous success towards greatly improving our understanding of the cellular pathology of molecular events associated with Alzheimer’s, as well as other neurodegenerative diseases. Of these, one of the most flexible, tractable, and versatile models has been the yeast *Saccharomyces cerevisiae *20,21,22,23,24. One of the things that make yeast such an important model for studying aggregation toxicity is that aggregation-prone, disease-associated proteins that would normally kill any mammalian cell are usually only mildly toxic in yeast. This suggests that yeast have efficient mechanisms for avoiding the aggregate toxicity that many human cells, and especially neurons, eventually succumb to. These mechanisms can be investigated in yeast with the hope of eventually exploiting them to address human pathology.

Another enormous advantage of yeast is the ease with which they can be used for high-throughput and high-content screening of genetic components of disease-associated molecular processes, as well as small molecule modulators of toxicity. Using a variety of high-throughput screening approaches several groups have identified genetic modifiers of α-synuclein toxicity 21,25, ALS-associated pathology 26, as well as Alzherimer’s-associated aggregation 27. Similarly, a number of promising small molecular modifiers of Parkinson’s cellular pathology 20 have been discovered through yeast screening.

**Figure 1:**
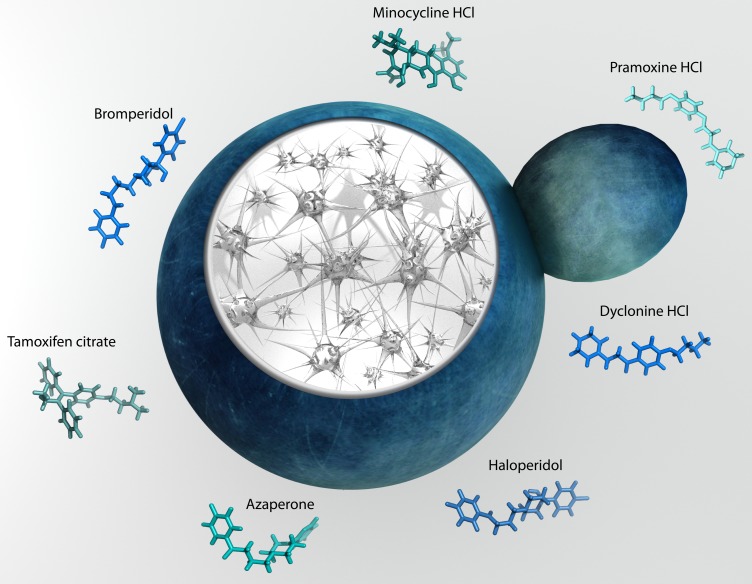
FIGURE 1: Yeast provide insight into molecular pathogenesis of Alzheimer disease and reveal modifiers of amyloid beta toxicity.

Recently, Park and colleagues working in Susan Liebman’s group discovered a number of small molecules capable of modulating Aβ aggregation in a yeast model 28. The group constructed an Aβ fused to a translation factor domain to assess the Aβ oligomerization with a simple growth assay 29. Park *et al*. screened 1200 FDA-approved drugs for the effect on Aβ oligomerization using a novel approach developed by the group earlier 28. The study uncovered 7 well-known compounds able to reduce oligomerization and rescue cellular toxicity in yeast (Fig. 1). These molecules were then shown to alleviate the toxic effects of Aβ aggregation in cultured mammalian cells, partially validating the yeast screen hits.

The molecules identified by the Liebman group show promise in that their molecular mechanism seems to involve modulating the small oligomers, thought to be the toxic species in the long chain of events leading to Aβ toxicity in the brain 28. All of the molecules identified have already been approved by the FDA for use in humans, significantly accelerating any potential drug-to-market timeline. The next step would be a direct test in an animal model of Alzheimer’s disease. In a promising precedent, a previous study describing novel targets for Parkinson’s disease in yeast has already proven to rescue neurons 21, illustrating the similarity and conservation in cellular response to amyloid aggregation, and the nearly unlimited utility of budding yeast to humanity^30^.
